# Evaluation of reliability and validity of the General Practice Physical Activity Questionnaire (GPPAQ) in 60–74 year old primary care patients

**DOI:** 10.1186/s12875-015-0324-8

**Published:** 2015-09-02

**Authors:** Shaleen Ahmad, Tess Harris, Elizabeth Limb, Sally Kerry, Christina Victor, Ulf Ekelund, Steve Iliffe, Peter Whincup, Carole Beighton, Michael Ussher, Derek G. Cook

**Affiliations:** Population Health Research Institute, St George’s University of London, Cranmer Terrace, SW17 0RE UK; Pragmatic Clinical Trials Unit, QMUL, London, UK; Department of Clinical Sciences, Brunel University, PO Box 4014, 0806 Oslo, Norway; Department of Sport Medicine, Norwegian School of Sport Sciences, PO Box 4014, 0806 Oslo, Norway; MRC Epidemiology Unit, University of Cambridge, Cambridge, UK; Research Department of Primary Care and Population Health, UCL, London, UK; Faculty of Health and Social Care, Kingston University, London, UK

**Keywords:** Health promotion, Public Heath, Primary health care, Questionnaire, Physical activity, Exercise, Walking, Ageing, Reliability, Validity

## Abstract

**Background:**

GPPAQ (General Practice Physical Activity Questionnaire) is a self-assessment physical activity questionnaire widely used in primary care. Reliability and validity data in older people are lacking.

The study aims were: to assess GPPAQ’s reliability and validity in 60–74 year olds from the PACE-Lift (Pedometer Accelerometer Consultation Evaluation-Lift) physical activity trial; and to assess whether adding brisk walking to the GPPAQ score improves its validity when assessing if physical activity guidelines are being met.

**Method:**

Physical activity was assessed objectively by accelerometry and by self-report GPPAQ over one week periods at baseline, and three and twelve months later, in 60–74 year old participants from three United Kingdom general practices enrolled in PACE-Lift. Reliability: GPPAQ scores in controls (n = 148) were compared for repeatability at baseline, 3 and 12 months. Validity: we compared the GPPAQ “active” rating (those not requiring physical activity advice) with those achieving physical activity guidelines using accelerometry, in all baseline subjects (n = 298). Using accelerometry as an objective comparator, GPPAQ sensitivity and specificity were calculated and repeated after adding brisk walking into the GPPAQ score (GPPAQ-WALK).

**Results:**

For reliability, GPPAQ showed 56 % (70/126) and 67 % (87/129) of controls scored the same at 3 and 12 months respectively, as they scored at baseline. At baseline 24 % (69/289) achieved physical activity guidelines according to accelerometry, whilst 16 % (47/289) were classified as GPPAQ “active”. GPPAQ had 19 % (13/69) sensitivity and 85 % (186/220) specificity. GPPAQ-WALK had 39 % (27/69) sensitivity and 70 % (155/220) specificity.

**Conclusions:**

GPPAQ has reasonable reliability but results from this study measuring validity in older adults indicates poor agreement with objective accelerometry for accurately identifying physical activity levels. Including brisk walking in GPPAQ increased sensitivity, but reduced specificity and did not improve overall screening performance. GPPAQ’s use in National Health Service health checks in primary care in this age group cannot therefore be supported by this validity study comparing to accelerometry.

**Electronic supplementary material:**

The online version of this article (doi:10.1186/s12875-015-0324-8) contains supplementary material, which is available to authorized users.

## Background

The health benefits of physical activity (PA) in older adults have been well documented with reports of significant reductions in morbidity and mortality from physical and mental health problems [1]. There is potential financial gain from increasing PA at a population level, with possible savings of £0.9 billion for the National Health Service (NHS) if physical activity targets are met [2]. It has been suggested that brief advice on PA should target the older population (>50y) as this has been shown to be more cost effective [3]. The Chief Medical Officer recommendations for PA [1] are reported in the 2013 NICE (National Institute for Clinical Excellence) guidelines for PA activity in adults which recommends a minimum of either ≥150 min of moderate to vigorous physical activity (MVPA) or ≥75 mins of vigorous physical activity (VPA) per week and suggests using the General Practice Physical Activity Questionnaire (GPPAQ) to identify those not meeting the guidelines, who would benefit from a brief PA intervention [4].

The GPPAQ is a relatively new addition to general practice and is used as a brief measure of PA in patients aged 16–74 years. It is designed to take less than one minute to complete and it groups subjects into 4 categories: inactive, moderately inactive, moderately active and active. It is either self-administered or completed with a healthcare professional and designed to form a basis for discussion and motivational interviewing for anyone who scores less than ‘active’ (*i.e.* assumed not to be meeting PA guidelines [4]). GPPAQ was commissioned by the Department of Health in 2006, developed by the London School of Hygiene and Tropical Medicine (LSHTM) [5] and it forms part of the NHS Health Checks, a routine check of cardiovascular health in general practice [6]. GPPAQ was included in the 2013/14 hypertension Quality and Outcomes Framework (QOF) (a pay for performance system for United Kingdom general practitioners) to incentivise the recording of PA and PA interventions, as discussed in the ‘Let’s get moving’ commissioning document [7]. It is derived from the short PA questionnaire used in the European Prospective Investigation into Cancer (EPIC) [8]. The LSHTM study to assess validity of GPPAQ took place in four UK GP (General Practitioner) practices with 334 patients aged 18–74 years (of whom only 43 were aged 65–74). Two hundred fifty eight completed a second GPPAQ questionnaire a week later and an unknown proportion wore Actigraph Motion sensors (accelerometers) for validation, (data unpublished). The NHS document on GPPAQ [5] refers to this validation work stating that GPPAQ has good face and construct validity and reliability and relates criterion validity to the original EPIC [9] study, however we were unable to find any published GPPAQ reliability or validity data.

GPPAQ scoring is limited to questions about occupation, cycling and sport. Retired or non-working respondents who do not do a sport or cycle cannot ever be classed as “active” and will always apparently warrant a PA intervention. Questions about walking and walking pace are included, but not scored in GPPAQ, although as scoring is done automatically by many GP computer systems, practitioners may not be directly aware of this. As walking is the main PA in older adults [10], this has implications for the validity of GPPAQ in this group. This has been highlighted as a concern of GPs in the ‘My Best Move’ Project [11] and discussed in the House of Lords Science and Technology Committee [12]. The 2013 NICE guidelines for PA acknowledged GPs’ concerns about GPPAQ but concluded that it would continue to support its use as it is a validated tool, but encouraged further studies [4]. GPPAQ remains a recommended tool in the NHS health check [6] but has been removed from the general practice 2014/15 hypertension QOF [13].

The PACE-Lift walking intervention trial measured PA using GPPAQ and objective accelerometer PA assessment in 60–74 year old primary care patients [14]. This paper uses PACE-lift trial data to address the following aims: to assess the reliability (repeatability) and validity of GPPAQ as a tool for measuring PA levels in 60–74 year olds using accelerometry as an objective comparator; to compare reliability of GPPAQ recorded routinely in primary care with trial GPPAQ recording; and to determine the effects of retirement status and inclusion of brisk walking on GPPAQ’s validity.

## Methods

### PACE-Lift trial methods

**Fig. 1:**
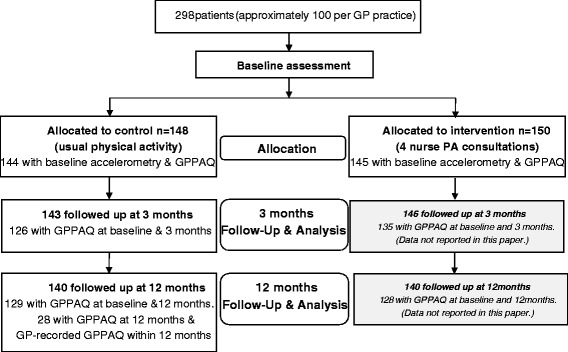
Modified from PACE-Lift Protocol [14]. All participants wore an accelerometer for 7 days and completed the GPPAQ questionnaire at each time point (baseline, 3 months and 12 months)

The PACE-Lift trial aimed to increase PA, particularly walking at moderate intensity, in older primary care patients (60–74 years) from three Berkshire and Oxfordshire UK practices using a complex intervention including pedometer, accelerometer, diary, nurse consultation and behaviour change techniques [14]. The trial had good recruitment for a PA intervention (298/988, 30 %) and participants wore the accelerometer on a belt around their waist, above their hip, for 7 days from rising in the morning until retiring to bed (excluding bathing or swimming) prior to completing GPPAQ at baseline (which asks about PA over the last 7 days). They were then randomly allocated into control and intervention groups. Both groups had accelerometry and GPPAQ repeated at three and twelve months (again, covering identical 7 day periods). Figure 1 shows the two study arms and PA data collection points.

### Physical activity assessment in PACE-Lift

i) Objective assessment

The Actigraph GT3X+ accelerometer records acceleration in 3 axes [15]. ActiGraph data were reduced using Actilife software set to derive 5 s epochs and to ignore runs of ≥60 min of zero counts [14, 16]. Only days with ≥540 min of wear time were included. The summary variables for the analysis were: counts per minute (CPM); time spent in at least moderate-to-vigorous PA (MVPA) (≥1952 CPM) and time spent in vigorous PA (VPA) (≥5725 CPM) levels using standard Freedson cut-point [17] and time spent in ≥10-min bouts of MVPA or VPA; we allowed for two minutes interruption (when CPA fell below the MVPA threshold) before the bout was deemed to have ended. This is the recommended default setting in the Actilife software. We used accelerometer data to categorise whether an individual achieved the PA guidelines (≥150 min of MVPA or ≥75 min of VPA, both in ≥10 min bouts, weekly) [4]. Accelerometry overcomes both recall and reporting bias and indicates whether moderate to vigorous intensity PA (MVPA) is accrued in bouts of ≥ 10 min, thus giving more accurate estimates of adherence to guidelines than self-report [18]. Accelerometry was used as the criterion method and it has been used successfully previously in older people with the same or very similar cut-points for MVPA and VPA [16, 18–20].

ii) Self-report questionnaire

**Fig. 2:**
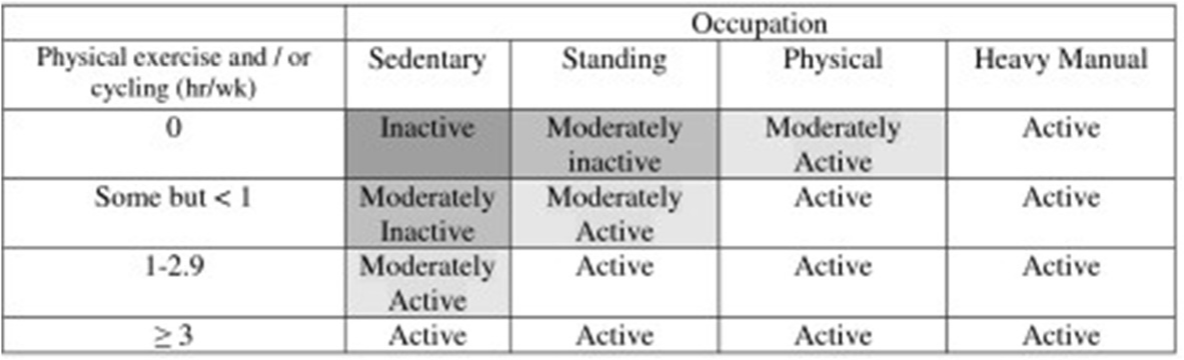
Summary of the GPPAQ Physical Activity Index Scoring [5]

The GPPAQ is a short self-report questionnaire on PA and occupation and is scored into active, moderately active, moderately inactive or inactive categories [5]. Active is taken as consistent with achieving PA guidelines relating to time spent in MVPA or VPA; all other categories require a PA intervention [5]. The GPPAQ questionnaire is included as an additional file 1 and Fig. 2 describes the scoring algorithm for GPPAQ.

### GPPAQ scores from computerised primary care records

As part of the trial, consent was sought to download information from participants’ computerised primary care records for the 12 month period of trial follow-up. If participants had a GPPAQ score recorded in their records during this time, as part of their routine care, it was downloaded (*n* = 56).

### Ethical review

The PACE-Lift trial was approved by Oxfordshire Research Ethics Committee C, UK (11/H0606/2), this approval gave permission for researchers from St George’s University of London to access the trial data. Written individual informed consent was obtained before trial procedures were performed.

### GPPAQ study methods

#### Reliability (repeatability)

The GPPAQ scores for each participant in the control group (in whom PA levels were not anticipated to change) were taken at baseline and three months and checked for agreement (n = 126). Baseline and 12 month GPPAQ scores were also compared to remove effects of seasonal variation (*n* = 129). GPPAQ scores from participants’ computerised primary care records (*n* = 56) were compared to PACE-Lift trial recorded GPPAQ scores, using trial data closest in time to the primary care record. For reliability, we have shown agreement for the two different time periods. We have also calculated the weighted kappa statistic for the four-category PA index from the baseline and follow-up questionnaires using an identical approach to that used in EPIC [8], with weights defined as 1-[(i-j)/(k-1)]^2^.

#### Validity

GPPAQ scores were divided into ‘active’ (considered to be achieving PA guidelines) or ‘less than active’ (comprising inactive, moderately inactive or moderately active). Objective accelerometry results were categorised into those meeting the guidelines (ie. achieving ≥150 min of MVPA or ≥75 min of VPA in ≥10 min bouts) classed as ‘active’ and the rest ‘inactive’. A 2x2 table was constructed comparing GPPAQ results for all individuals at baseline with their accelerometer results as gold standard (*n* = 289), and sensitivity and specificity were calculated. Sensitivity was defined as: the ability of GPPAQ to correctly identify as active those achieving the PA guidelines as assessed by accelerometry. Specificity was defined as: the ability of GPPAQ to correctly identify as not active those not achieving the PA guidelines by accelerometry.

#### GPPAQ-WALK

Questionnaires from all at baseline (*n* = 289) were re-coded to include walking in the scoring (GPPAQ-WALK). GPPAQ-WALK differed from GPPAQ in that participants who reported walking at a brisk or fast pace for ≥3 h/week were also re-coded as active. This cut-off for time spent walking was chosen as it is the pre-specified cut-off on the GPPAQ questionnaire (Fig. 2) which corresponds to those who definitely self-report walking at a brisk or fast pace for long enough to achieve the PA guidelines by walking alone [1]. The validity (sensitivity and specificity) of GPPAQ-WALK was then calculated as for GPPAQ.

#### Effect of employment on GPPAQ validity

The validity of GPPAQ at baseline in participants who were not in employment (*n* = 193) was compared to those in employment (*n* = 105) to see if this affected its sensitivity and specificity.

#### Distribution of MVPA by GPPAQ score

We calculated the mean weekly minutes of MVPA in 10 min bouts and the proportion achieving the recommended guidelines by the four GPPAQ and GPPAQ-WALK categories.

## Results

Table 1 shows characteristics of the PACE-Lift participants. All age groups were well represented (60-64y, 65-69y and 70-74y) and approximately half the participants were men. The majority were retired, married and in good health, had low levels of chronic disease and disability and were mainly overweight or obese. The average daily wear-time was 793 min (mean), with a standard deviation of 75.7 min, median of 791 min and range of 615–1019 min.

**Table 1:** Baseline characteristics of the Pace-Lift cohort

	*N* = 298 n (%)
Age at randomisation^1^	
60-64 years	110 (37 %)
65-69 years	105 (35 %)
70-74 years	83 (28 %)
Gender: Male	138 (46 %)
Retired/not in employment (GPPAQ questionnaire)	193 (67 %)
Marital status: married	240 (81 %)
General Health^2^: Very Good or Good	260 (89 %)
Chronic diseases^2^	
None	91 (31 %)
1-2	178 (60 %)
≥3	29 (10 %)
Townsend disability score^2^	
None (0)	204 (70 %)
Mild disability (1–5)	82 (28 %)
Moderate or severe disability (6–18)	7 (2 %)
Body Mass Index: Overweight/obese (≥25 kg/m^2^)	200 (67 %)
Accelerometry data (mean (sd) of adjusted data^3^):	
Baseline step count per day	7347 (2839)
Total weekly minutes of moderate or vigorous physical activity (MVPA) in bouts of at least 10 min	92 (108)

^1^10 subjects were 75 years at randomisation, due to delays between invitation to participate and randomisation

^2^Full references for General Health, Chronic Disease Score and Townsend Disability Score, are given in the trial protocol [14]

^3^Accelerometry data (step counts and MVPA) were adjusted for day of the week and day order of wearing the accelerometer in a multi-level model with household and participant as random effects

The average daily step-count at baseline was 7347 (sd 2839) (Table 1) and the average weekly MVPA in bouts of ≥10 mins was 92 (sd 108), well below recommended guidelines.

### Reliability (Table 2)

**Table 2:** Assessing the repeatability of the GPPAQ questionnaire data Pace-Lift GPPAQ at baseline, 3 months and 12 months

GPPAQ at baseline	Inactive	Moderately Inactive	Moderately active	Active	Total	Repeatability	Weighted Kappa^1^
GPPAQ at 3 months							
Inactive	44	10	5	2	61		
Moderately inactive	6	5	5	2	18		
Moderately active	11	1	9	8	29		
Active	1	2	3	12	18		
3 month totals	62	18	22	24	126	70/126 = 56 %	0.57
GPPAQ at 12 months							
Inactive	56	8	9	3	76		
Moderately inactive	6	4	2	2	14		
Moderately active	3	3	9	2	17		
Active	0	1	3	18	22		
12 month totals	65	16	23	25	129	87/129 = 67 %	0.63
Pace-Lift 12 month GPPAQ and GPPAQ within 12 months extracted from GP records							
Pace-Lift GPPAQ (self-administered at 12 months)							
	Inactive	Moderately inactive	Moderately active	Active	Total	Repeatability	
GPPAQ extracted from GP records							
Inactive	9	1	1	0	11		
Moderately inactive	3	1	1	2	7		
Moderately active	3	0	1	0	4		
Active	3	1	0	2	6		
Totals	18	3	3	4	28	13/28 = 46 %	0.24
1. Weighted Kappa calculated using weights (1.0, 0.8889, 0.5556, 0)							

There were 148 participants in the control group at baseline and 143 at three months, 126 had the GPPAQ recorded at both time points. Table 2 shows that the percentage of GPPAQ scores that were the same (agreement) is 56 % (70/126) at three months (weighted kappa 0.57) and 67 % (87/129) at 12 months (weighted kappa 0.63).

### Comparison of GP and trial recorded GPPAQ data

Activity levels of participants in the control arm with a GP recorded GPPAQ administered during the 12 month trial period were compared with GPPAQ recorded at 12 months as part of the study (n = 28) and showed a 46 % agreement (weighted kappa 0.24).

### Validity (Table 3)

**Table 3:** Assessing the validity of GPPAQ questionnaire data using accelerometry data as the “gold standard”

		All *N* = 289	Retired/Not working *N* = 193	Working *N* = 96
Baseline accelerometry				
Those achieving DH Physical Activity guidelines of 150 min of MVPA in 10 min bouts^1^		69 (24 %)	50 (26 %)	19 (20 %)
Baseline GPPAQ Physical Activity Index				
Not active:	GPPAQ Inactive	152 (53 %)	116 (60 %)	36 (38 %)
	GPPAQ Moderately inactive	35 (12 %)	18 (9 %)	17 (18 %)
	GPPAQ Moderately active	55 (19 %)	29 (15 %)	26 (27 %)
Active:	GPPAQ Active	47 (16 %)	30 (16 %)	17 (18 %)
Sensitivity of GPPAQ to identify “Active” individuals		19 % (13/69)	18 % (9/50)	21 % (4/19)
Specificity of GPPAQ to identify “Not active” individuals		85 % (186/220)	85 % (122/143)	83 % (64/77)
Baseline GPPAQ-Walk Physical Activity Index^2^				
Not active:	GPPAQ Inactive	123 (43 %)	97 (50 %)	26 (27 %)
	GPPAQ Moderately inactive	30 (10 %)	15 (8 %)	15 (16 %)
	GPPAQ Moderately active	44 (15 %)	20 (11 %)	24 (25 %)
Active:	GPPAQ Active	92 (32 %)	61 (32 %)	31 (32 %)
Sensitivity of GPPAQ-walk to identify “Active” individuals	39 % (27/69)	40 % (20/50)	37 % (7/19)	
Specificity of GPPAQ-walk to identify “Not active” individuals	70 % (155/220)	71 % (102/143)	69 % (53/77)	

1. DH Guidelines recommend at least 150 min of MVPA in bouts of at least 10 mins

2. GPPAQ-Walk additionally classifies those who walk ≥3 h per week at a “brisk” or “fast” pace as active

Two hundred eighty nine of the 298 participants at baseline had both GPPAQ scores and accelerometry. Using accelerometry to assess PA, 24 % (69/289) of participants met current guidelines, whereas only 16 % (47/289) were classed as “active” according to GPPAQ. Sensitivity of the GPPAQ was 19 % (13/69) and specificity 85 % (186/220) compared to accelerometry.

### GPPAQ-WALK

The percentage classified as active by GPPAQ-WALK was 32 % (92/289). Including walking in the scoring changed the validity. GPPAQ-WALK had a sensitivity of 39 % (27/69) and a specificity of 70 % (155/220).

### Effect of employment

Table 3 shows that in standard GPPAQ, sensitivity for detecting truly active subjects is marginally higher in those who are working than in those who are not in employment, 21 % vs 18 %. This is reversed in GPPAQ-Walk where sensitivity in those who work is 37 % and in those who are not in employment is 40 %.

### Weekly MVPA and proportion achieving PA guidelines by GPPAQ & GPPAQ-WALK categories (Table 4)

**Table 4:** Comparison of GPPAQ questionnaire information and MVPA from accelerometry

		Total weekly MVPA in 10 min bouts	Achieved DH Guidelines for physical activity^1^
Baseline standard GPPAQ Physical Activity Index	N	Mean (sd)	N (%)
Inactive	152	91 (112)	36 (24 %)
Moderately inactive	35	84 (91)	8 (23 %)
Moderately active	55	96 (111)	12 (22 %)
Active	47	99 (111)	13 (28 %)
Baseline standard GPPAQ-WALK Physical Activity Index^2^			
Inactive	123	80 (97)	27 (22 %)
Moderately inactive	30	85 (93)	6 (20 %)
Moderately active	44	86 (108)	9 (20 %)
Active	92	115 (126)	27 (29 %)

1. DH Guidelines recommend at least 150 min of MVPA in bouts of at least 10 mins

2. GPPAQ-Walk additionally classifies those who walk ≥3 h per week at a “brisk” or “fast” pace as active

Looking at mean total weekly MVPA in 10 min bouts there is no clear pattern across the four categories of standard GPPAQ. There is however a clear trend in MVPA in GPPAQ-WALK: active > moderately active > moderately inactive > inactive. The difference is less clear when looking at the percentage achieving PA guidelines, where there is no clear trend in either group.

## Discussion

### Main findings

GPPAQ is acceptable to older primary care patients, has reasonable reliability, particularly when repeated in the same season, with 67 % agreement in findings at 12 months. However, it has poor validity in this age group for identifying PA levels accurately, with a sensitivity of only 19 % compared to objective accelerometry PA assessment. The sensitivity was more than doubled by including brisk walking in the scoring (GPPAQ-Walk), but at the cost of a marked reduction in specificity, suggesting that modifying GPPAQ to include walking in this age group did not improve its overall performance as a screening test.

### Strengths and limitations

#### Strengths

This study has a large number of participants and also has an objective PA measure, accelerometry, as the criterion, with which GPPAQ was compared. Accelerometry was administered over the same 7 day period preceding the completion of GPPAQ, so the PA measurements correspond to the time period covered by the questionnaire. Evidence from average wear-time indicated that there was a high average wear-time (over 13 h per day, which covers approximately 8 am to 9 pm) and indicated that we captured a high proportion of the physical activity achieved by this population. We have repeated measures of GPPAQ at baseline, 3 months and 12 months and also from computerised primary care records, allowing comparisons over a short time period (but different season) and longer time period (but same season) as well as with data collected in different ways by different individuals. We have data on occupational status/retirement, allowing us to examine the effect of this on GPPAQ validity. We have examined the effect on validity of adding in brisk or fast walking to the GPPAQ score, as this is the primary PA in older people. The participants are primary care patients, the target group for GPPAQ, approximately half are male and they are comparable in terms of the proportion who are overweight and obese with a recent population based survey of this age group (68 % in PACE-Lift, 73 % in Health Survey England [21]).

#### Limitations

The Actigraph GT3X+ accelerometer does not register complex movements well, consequently power sports (such as weight lifting), swimming and cycling are not accurately registered [22]. However, it monitors walking, the main PA in older adults, accurately, is able to identify if PA is occurring in ≥10 min bouts, as stipulated by guidelines, is widely used in PA research [23]. There are small amounts of missing GPPAQ data: (9/298 (3 %) at baseline, 31/298 (10 %) at three months and 16/298 (5 %) at twelve months). However, this is unlikely to bias analyses which are all within person comparisons. The pre-specified cut-off on time spent walking used to define GPPAQ-WALK (≥3 h weekly) was not exactly the same as those used in guidelines (2.5 h weekly), but was the nearest provided by the questionnaire. The effect of using a slightly higher, more difficult to achieve cut-off (≥3 h rather than ≥2.5 h) means that we may have underestimated the sensitivity and overestimated the specificity. However, GPPAQ-WALK already demonstrated greater sensitivity but poorer specificity than GPPAQ, so the direction of any effect of using the lower cut-off of ≥ 2.5 h would be unchanged from what we have described. The PACE-Lift trial only includes 60–74 year olds and is therefore not able to comment on GPPAQ’s reliability and validity in 40–59 year olds, where its use is also promoted in NHS health checks [6].

### Comparison with existing literature

Other studies have used correlation coefficients for questionnaire validation [8]. However, this relies on arbitrary weighting decisions. We chose to present the raw data and calculate percentage agreement for reliability in addition to kappa statistics and to present sensitivity and specificity for validity (as has also been presented by others [24]). The limitations of using correlation coefficients in PA questionnaire assessment have been discussed in other papers where Bland-Altman plots have been chosen instead [25]. As described previously, there are no published studies with data on GPPAQ’s reliability and validity, and this is the first study to assess GPPAQ against objective accelerometer assessment in a large sample of older adults. GPPAQ is derived from the EPIC PA questionnaire (EPIC-PAQ), which has been validated (good reliability, weighted kappa = 0.6, p < 0.0001 and positive associations with objective measures of energy expenditure, p = 0.003) [8]. Though GPPAQ was derived from EPIC-PAQ, it is difficult to use data on validation of EPIC-PAQ to throw light on our findings as their validation was primarily focussed on demonstrating correlations between accelerometry and their questionnaire measures, and not on the sensitivity and specificity for identifying those requiring intervention to increase PA [26] [9]. EPIC-PAQ relies on recall of PA over the last year whereas GPPAQ relies on recall over the preceding week, and EPIC-PAQ was validated in 50–65 year olds, whilst GPPAQ is aimed at 16–74 year olds [5] and used in NHS health checks for 40–74 year olds [6].

A feasibility study of GPPAQ use in patients aged 35–74 years across four Northern Ireland general practices examined 192 questionnaires and found that GPs and nurses reported it was an easy tool to assess PA levels with, although integration within routine practice was limited by time constraints and complex consultations [27]. An important study limitation was the 8 % GPPAQ completion rate [27].

In terms of other short PA self-report measures which could be appropriate for primary care, a systematic review of studies validating the short form of the International Physical Activity Questionnaire (IPAQ) found that it significantly overestimated PA when compared to objective measurement [28]. A recent review of reliability and validity of 34 new PA questionnaires assessed their performance across age groups. In the elderly they found that although there is a reasonable reliability (median correlation coefficient 0.60-0.65 in existing questionnaires and 0.70 in the newer questionnaires) the validity was ‘poor to acceptable’ in the elderly (0.35-0.40 in existing and 0.41 in new questionnaires). They also identified sedentary behaviour as a difficult domain to assess using questionnaires, with poor correlation with objective measures [29]. A global physical activity questionnaire that has been validated against national self-report survey data in a large study of almost 2 million patients in the US is the Exercise Vital Sign- a 2-item PA questionnaire [30] but to date has not been validated against an objective PA measure or used in UK primary care.

### Implications for research and practice

The health benefits of PA, specifically in older adults, have been well documented and an accurate validated tool that would identify which older patients would benefit most from a PA intervention would be of great benefit in general practice. In response to GPs’ concerns over GPPAQ, NICE recommended further studies. This study suggests that whilst the GPPAQ has reasonable reliability, it is not a valid tool for assessing PA levels in older adults. Our findings therefore support the retraction of GPPAQ from the hypertension QOF [13] and question its continued use in NHS health checks [6] for this age group. Currently while there are good cheap pedometers which, when worn on the hip measure step-counts, they do not measure physical activity intensity. Accelerometers worn on the hip, such as those used in our study, provide measures of both steps and intensity and thus of MVPA. They are however relatively expensive and a little uncomfortable to wear. Wrist worn devices offer improved wear acceptability, and potentially 24 h wear-time, and can be waterproofed. However, accelerometers worn on the hip generally do better than similar data from wrist worn accelerometers for measuring energy expenditure. Moreover, accurately identifying sedentary behaviour from a lack of wrist motion presents significant challenges [31]. Nevertheless, improvements and rapid technological advances in PA measurement, including the use of smartphone applications and cheap accelerometers, are likely to provide more robust measures of PA in primary care, rather than relying on short but invalid questionnaires.

## Conclusions

This paper using data from a primary care physical activity trial in 60–74 year olds is the first to provide published validation evidence on GPPAQ, a widely used assessment tool in NHS primary care. The reliability (repeatability) was reasonable with 67 % agreement at 12 months, but the validity was poor, with 19 % sensitivity and 85 % specificity compared to accelerometry. Overall screening performance was not improved by adding brisk walking to the GPPAQ score.

Our findings support the retraction of GPPAQ from the GP hypertension QOF and question its continued use in NHS health checks in this age group. Rapid technological advances in PA measurement, including the use of smartphone applications and cheap accelerometers, are likely to provide more robust measures of PA in primary care, rather than relying on short but invalid questionnaires. Further work is needed to identify an accurate validated tool that would identify which older patients would benefit most from a PA intervention within primary care.

## Additional file

Additional file 1:
**General practice physical activity questionnaire.** (JPEG 87 kb)

## Abbreviations

PA: Physical activity
NHS: National Health Service
NICE: National Institute for Clinical Excellence
MVPA: Moderate to vigorous physical activity
VPA: Vigorous physical activity
GPPAQ: General Practice Physical Activity Questionnaire
LSHTM: London School of Hygiene and tropical medicine
QOF: Quality and outcomes framework
EPIC: European Prospective Investigation into Cancer
GP: General practitioner
PACE-Lift: Pedometer accelerometer consultation evaluation-lift
EPIC-PAQ: EPIC PA questionnaire
IPAQ: International Physical Activity Questionnaire
