# Supplementary figures and images for: Evaluation of reliability and validity of the General Practice Physical Activity Questionnaire (GPPAQ) in 60–74 year old primary care patients
Source: BMC Fam Pract. 2015 Sep 2;16:113. doi: 10.1186/s12875-015-0324-8 (PMC4557746; doi:10.1186/s12875-015-0324-8)

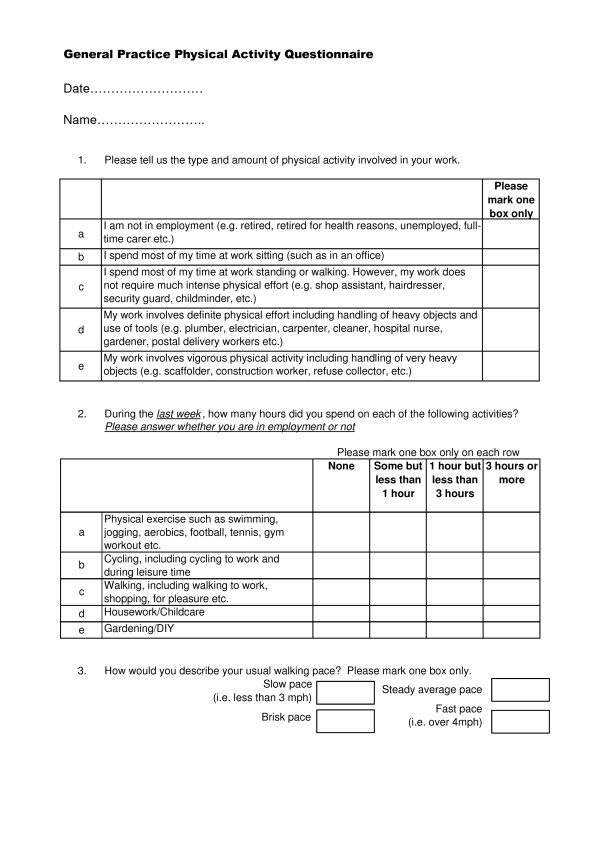

Supplement: Additional file 1: — General practice physical activity questionnaire. (JPEG 87 kb) [file 12875_2015_324_MOESM1_ESM.jpeg]
